# In young women, a link between childhood abuse and subliminal processing of aversive cues is moderated by impulsivity

**DOI:** 10.1186/s12888-022-03770-0

**Published:** 2022-03-02

**Authors:** P. S. Regier, L. Sinko, K. Jagannathan, S. Aryal, A. M. Teitelman, A. R. Childress

**Affiliations:** 1 Perelman School of Medicine, Department of Psychiatry, University of Pennsylvania, Philadelphia, PA USA; 2 School of Nursing, University of Pennsylvania, Philadelphia, PA USA; 3 College of Public Health, Temple University, Philadelphia, USA

**Keywords:** Childhood maltreatment, fMRI, Impulsivity, Negative cues, Mental health

## Abstract

**Background:**

Childhood maltreatment is a serious public health concern. The association between child maltreatment, adverse behaviors, mental health outcomes, and alterations to brain function and structure have begun to be characterized. Less is known about the specific associations of maltreatment subtypes with cue-response to evocative cues and the moderating effects of confounding mental health/behavioral variables.

**Methods:**

Fifty-four emerging adult women (aged 18–24) completed assessments for behaviors, mental health, and childhood maltreatment. They participated in a fMRI task featuring passive viewing of evocative (33 ms) cues presented by “backward masking” to prevent conscious processing. Correlations of abuse/neglect scores, behavioral/mental health factors, and brain function were assessed. Follow-up analyses investigated the moderating effects of behavioral/mental health factors on maltreatment and brain relationships.

**Results:**

Greater frequency of childhood abuse and neglect were correlated with higher scores of impulsivity, depressive symptoms, and anxious attachment. Childhood abuse was positively associated with increased medial orbitofrontal cortical (mOFC) response to aversive (vs. neutral) cues. Among the behavioral/mental health variables, only impulsivity appeared to have a moderating effect on the relationship between childhood abuse and brain response to aversive cues.

**Conclusions:**

The link between childhood abuse and a heightened mOFC response to “unseen” aversive stimuli, moderated by impulsivity, adds to the growing literature on the impact of prior adversity on brain function. These findings offer further understanding for the way in which childhood maltreatment affects the brain processing of negative stimuli, helping to explain the well-documented link between childhood maltreatment and a variety of adverse outcomes in adulthood.

**Supplementary Information:**

The online version contains supplementary material available at 10.1186/s12888-022-03770-0.

## Introduction

Childhood maltreatment is a serious public health concern across the globe. Maltreatment is generally classified by four main subtypes: physical abuse, sexual abuse, emotional abuse, and neglect [1], with prevalence rates ranging internationally from 16.6 to 36.3% [2]. In the United States (U.S.), it is estimated that rates are similar to global estimates at 14–29% [2], and data suggest 30% of individuals living in the U.S. have a history of at least one form of child maltreatment, with 14% reporting multiple forms of maltreatment [3]. In the U.S., girls disproportionately experience childhood abuse. For example, 1 in 5 women have a history of sexual abuse, and, notably, most recent data suggests sexual victimization is more than twice as high for girls compared to boys [4].

The association between childhood maltreatment and adverse health outcomes is well established [5–9], including childhood maltreatment’s impact on mental health and disadvantageous behaviors. For example, studies have found childhood maltreatment is associated with higher impulsivity [10], increased sexual risk behaviors [5], as well as a higher likelihood of insecure attachment, depression, anxiety, and substance-use disorder [9, 11, 12].

Experiences of excess or sustained stress can have lasting impact on long-term physiological, behavioral, and psychological health and well-being, particularly when it occurs early in life. In line with these data, the current study is guided by frameworks applying a life-course perspective, highlighting the importance of early-life experiences on health and disease as one ages and develops [13]. For instance, childhood maltreatment can alter brain development, impairing both brain architecture and brain function as an adult [14, 15], which has an effect on health issues later in life [16]. In particular, mental health disorders (e.g., depression) are common in those with childhood maltreatment [17, 18] and may interact with brain development. This study utilizes data from a previous study, whose focus was on emerging adult women (ages 18–24). This time-period for women represents a heightened vulnerability to abuse, and also a phase of continued brain development, particularly in fronto-limbic connections [19]. A greater understanding of brain-behavior relationships during this developmental period may inform future interventions early in the adult life course to mitigate the deleterious impact of child maltreatment.

At the level of the brain, research has shown differences of structure and function associated with maltreatment. For example, several studies have found that grey matter volume (GMV) is lower for individuals with higher levels of childhood maltreatment in brain regions important for memory [20–22], decision-making [23], emotional regulation [24], and reward processing [25], and data suggest maltreatment subtypes (e.g., emotional, physical, and sexual abuse; emotional and physical neglect) may be associated with structural abnormalities in different parts of the brain (e.g., GMV in amygdala; size of corpus collosum, for reviews, see [14, 15]). Childhood maltreatment is also associated with a differential brain response to salient information, such as threat detection and the promise of rewards [15]. For example, previous research in adults who had experienced childhood maltreatment revealed increased reactivity to traumatic scripts in cingulate, motor cortex, and frontal gyrus [26]. In addition, higher childhood maltreatment scores were associated with increased amygdala and/or hippocampus response to emotional faces, processed both at the conscious [27] and subconscious [28] level. In contrast, childhood maltreatment has been associated with a blunted response to reward anticipation [25, 29–31] with limited data suggesting an increased response to reward receipt [30]. Further data are needed to help elucidate the impact of maltreatment (and its subtypes) on the brain’s processing of negative and positive stimuli.

Similar to the effects of maltreatment, frequently occurring comorbid behavioral issues and mental health symptoms, such as depression and anxiety, separately are associated with a hyper-reactivity to negative stimuli and blunted response to rewarding stimuli [32, 33]. In contrast, higher impulsivity scores appear to be associated with a reduction in response to negative stimuli, but an increase to rewarding stimuli [34–37]. Because maltreatment and mental health/maladaptive behaviors are often correlated, there is a need to understand how these variables moderate [38] any observed relationship between childhood maltreatment and the brain response to evocative cues.

In this secondary analysis, we assessed childhood (physical, sexual, and emotional) abuse and (physical and emotional) neglect, creating total childhood abuse (TCAs) and neglect (TCNs) scores from a well-validated measure of childhood maltreatment, the Childhood Trauma questionnaire [39]. We also gathered mental health/behavioral data, such as depression, anxious attachment, sensation seeking, and impulsivity scores. After surveys and questionnaires, study participants underwent a brain MRI scan that captured GMV and then measured regional brain blood flow (an index of neural activity) while participants passively viewed images of aversive and appetitive (romantic and food) cues, as well as neutral comparator cues, processed at the subconscious (e.g., backward-masked 33 ms cues) [40, 41]. These subliminal cues are useful for probing the immediate brain response to evocative stimuli, ungoverned by conscious evaluation that could alter or confound the early brain response.

The aims of this study were: 1) to describe the relationships between TCAs and TCNs with mental health symptoms (i.e., depression, anxious attachment) and behavioral variables (i.e., impulsivity, sensation seeking); 2) to understand the brain response to subconsciously-processed evocative images as a function of TCAs and TCNs; and 3) to examine whether mental health symptoms and/or behavioral variables moderate the relationships between TCAs and/or TCNs and the functional brain response to the 33 ms cues. Structural (GMV) data was primarily used to replicate and confirm findings from previous studies (see above).

We hypothesized a heightened response in “cue-reactive” regions to (backward-masked) 33 ms aversive and (vs. neutral) cues correlated with higher TCAs and/or TCNs, since, as noted above, maltreatment has previously been associated with increased subcortical activity to threat and negative stimuli. The prior literature on childhood maltreatment with reward responding, (anticipation and reward) is mixed, but our own study of adult cocaine patients with a history of abuse found heightened brain response to drug reward cues [42]. This encouraged a prediction that childhood maltreatment would be positively correlated with the brain response to appetitive (romantic and/or food) cues. Finally, given the prior literature on links between childhood maltreatment and later behavioral and mental health symptoms, we hypothesized some (or all) of these variables would moderate the putative relationship between maltreatment and the brain response to evocative cues.

## Method and materials

This is a secondary analysis using data from the Behavior in Urban Female Risk Study (BUFR), which examined the neural response to neutral and evocative cues (food, romantic, aversive) in emerging adult women (ages 18–24) [43]. Participants (*n* = 60) were recruited from a family-planning clinic and from a nearby university, both of which were located in a large urban area in the northeast United States. Subjects participated in two sessions, typically scheduled on consecutive days. In the first session, participants completed informed consent, surveys, and demographic health information via pen and paper. For sensitive topics like intimate partner violence and adverse childhood experiences, Audio Computer Assisted Self-Interviewing (ACASI) was utilized for data collection in order to increase the accuracy of self-report data [44]. In the second session, a functional magnetic resonance imaging (fMRI) scan was completed while participants performed selected tasks on a computer. Due to the nature of fMRI, participants were excluded if they had a history of a seizure disorder, were greater than approximately 300 pounds, or were pregnant. Following completion of the study, participants were compensated 105 dollars. This study was approved by University of Pennsylvania institutional review board. The authors assert that all procedures contributing to this work comply with the ethical standards of the relevant national and institutional committees on human experimentation and with the Helsinki Declaration of 1975, as revised in 2008.

### Measures

#### Demographic and health variables

Participants were assessed for age, current education status, mother’s highest education, race (Caucasian/White, African American/Black, Native American, Asian/Pacific Islander, Other) and ethnicity (Hispanic/Latino). Participants were asked about substance use (cigarettes, alcohol, cocaine, marijuana) in their lifetimes and in the past 30 days. Two questions assessed food insecurity: 1) Do you ever have to make choices between spending money on food or spending money on other needs? (i.e. medication, utilities, rent, etc.); 2) Do you ever worry whether your food will run out before you have money to buy more?

#### Child maltreatment

The short form Childhood Trauma Questionnaire (CTQ-SF) [45] is a well-established modified version of the CTQ [39] that includes 28 (of the original 70) items that measures emotional, physical, and sexual abuse as well as emotional and physical neglect prior to the age of 16. Items are rated on a 5-point Likert scale ranging from 1 (never true) to 5 (very often true). Previous studies have utilized the CTQ to establish different abuse and neglect subcategories to examine their impact on grey-matter volume [46–48], Similarly, the present study summed total scores for childhood abuse (TCAs) (i.e. emotional, physical, sexual) and neglect (TCNs) (i.e., emotional, physical) separately, which were used for brain and behavioral analyses. Possible range for TCAs was 15 to 75; for TCNs the possible range was 10 to 50. Cronbach’s alpha for our sample was 0.94.

#### Depression

The Center for Epidemiological Studies-Depression (CES-D) questionnaire [49], is a 20-item measure that asks participants to rate how often over the past week if they experienced symptoms associated with depression, such as restless sleep, poor appetite, and feeling lonely. Response options range from 0 to 3 for each item (*rarely or none of the time, some or little of the time, moderately or much of the time, most or almost all the time*). Scores could range from 0 to 60, with high scores indicating greater depressive symptoms. Cronbach’s alpha for our sample was 0.74.

#### Impulsivity

The Barratt Impulsivity Scale (BIS) [50] is a 30-item questionnaire that measures impulsiveness through items such as “I act on impulse” and “I consider myself always careful”. Participants indicated how frequently each statement applies to them on a 1 to 4 point Likert scale (*never*, *occasionally*, *often*, *almost always*). Possible score totals ranged from 30 to 120, with higher scores indicating greater total levels of impulsiveness. Cronbach’s alpha for our sample was 0.87.

#### Anxious attachment

The present study utilized 4 out of the 6 questions from the Adult Attachment Scale’s anxiety dimension [51]. Two questions from the anxiety dimension were not used due to low item-total correlation [52]. Questions measured subjective attitudes of self and others in a romantic relationship, e.g., “I often worry that my romantic partner does not really love me.” Participants answer with a 1 to 5 point Likert scale (*disagree strongly, disagree, in the middle, agree, agree strongly*). Score totals could range from 5 to 20, with higher scores indicating higher levels of anxious attachment. Cronbach’s alpha for our sample was 0.64.

#### Sensation seeking

The Brief Sensation Seeking Scale [53] is an 8-question well-known adaptation of the Sensation Seeking Scale [54]. Questions assess thrill seeking, disinhibition, boredom susceptibility, and experience seeking, with questions, such as “I would like to explore strange places” and “I like wild parties”. Participants indicated whether how much they agree or disagree with a 1 to 5 point Likert scale (*disagree strongly, disagree, neither disagree nor agree, agree, and agree strongly*). Total scores could range from 8 to 40, with higher scores indicating greater levels of sensation seeking. Cronbach’s alpha for our sample was 0.67.

### Imaging

As described previously [43, 55], participants underwent a blood-oxygen-level dependent (BOLD) fMRI scan. Images were obtained via a Siemens 3 Tesla (Trio) research-dedicated magnet, which included an 8-channel head-coil, an LCD projector for stimulus presentation, air-conducting earphones, and a fiber optic response pad. Attached to the head coil were mirrors that could be adjusted so that participants could focus attention on stimuli and instructions projected on a computer screen. The scan session began with a 3-min localizer scan and a T1-weighted high-resolution scan (MPRAGE) was acquired. Subsequently, for functional scans, T2*-weighted BOLD images were obtained with single shot gradient echo planar imaging sequence (field of view = 192 mm, matrix 64 × 64, TR = 2 s, TE = 30 ms, flip angle = 80).

In the scanner, participants were shown 33 ms evocative (aversive, romantic, food) and neutral cues, followed by 467 ms of a neutral image, which functions as a backward mask. Participants in this procedure (i.e., backward masking) have reported seeing the longer stimulus, while the 33 ms target stimulus remains “unseen” [40]. Target (and mask) stimuli were interspersed with grey screens with a single crosshair presented at a random duration between 1000 ms and 2000 ms, with an average of approximately 1500 ms. The task consisted of two rounds of cue presentation. Twenty-four novel cues from each category were presented in pseudorandom order, followed by repeated presentation of those same cues. The food cues (e.g., images of desserts, pasta, French fries, “highly palatable foods”), neutral cues (household or office objects; outdoor scenes) were from laboratory archives. The aversive cues and more than half of the romantic cues were selected from the top quartile (e.g., “most unpleasant” and “most pleasant”, respectively) of the International Affective Picture System (IAPS, Lang et al., 1999 [56]). The remainder of the romantic cues were specifically generated to reflect greater racial diversity.

Previous childhood maltreatment studies with functional imaging probes used either scripts [20, 26], or emotional faces, presented inside [27] or outside, [28] awareness. Our use of aversive cues from the IAPS extends the childhood maltreatment-brain literature to this well-studied brain probe.

### Statistical methods

#### Behavioral and health data

To describe our dataset, we calculated mean and standard deviation for continuous variables and frequency distributions for categorical variables. Pairwise correlation analysis was conducted to evaluate association of TCAs and TCNs with continuous variables, and analysis with 2-sample t-tests was conducted to evaluate the difference of TCAs and TCNs between categorical groups. Confidence intervals and effect sizes were calculated and reported when appropriate. To account for multiple comparisons, we adjusted our significance level using False Discovery Rate (FDR) approach [57], using a custom-built function [58] via MATLAB (Mathworks, 2019a) [59].

#### Imaging data

##### First-Level Analysis

Data processing was carried out in SPM8 (http://www.fil.ion.ucl.ac.uk/spm) run under MATLAB R2019a, as previously described [43, 55]. Briefly, each participants’ images were slice-timing corrected, realigned, co-registered to high-resolution to structural images (MPRAGE), and subsequently normalized to MNI standard space and smoothed with the FWHM kernel of 9 mm [44]. The motion statistics for each subject were examined to ensure that motion did not exceed 2 mm in any plane. For the first level analysis, a canonical hemodynamic response function with time and dispersive derivatives was fitted to the onset of each event. The following first-level contrasts were defined to assess the cue effect: aversive vs. neutral, sexual vs. neutral, and food vs. neutral.

Data for structural grey matter volumes (GMV) was prepared for analyses as previously described [60], utilizing an external toolbox for SPM (VBM-DARTEL) as described in the VBM tutorial (http://www.fil.ion.ucl.ac.uk/~john/misc/VBMclass10.pdf). Preprocessing for VBM steps involved generating roughly aligned grey and white matter images, determining nonlinear deformations for warping grey and white matter images in order to match each other, and generating smoothed warped grey and white matter images. Voxel sizes of 1.5 × 1.5 × 1.5 was used for spatially normalized images, and the gaussian FWHM was set at 10 mm.

##### Second-Level Analysis

**Fig. 1:**
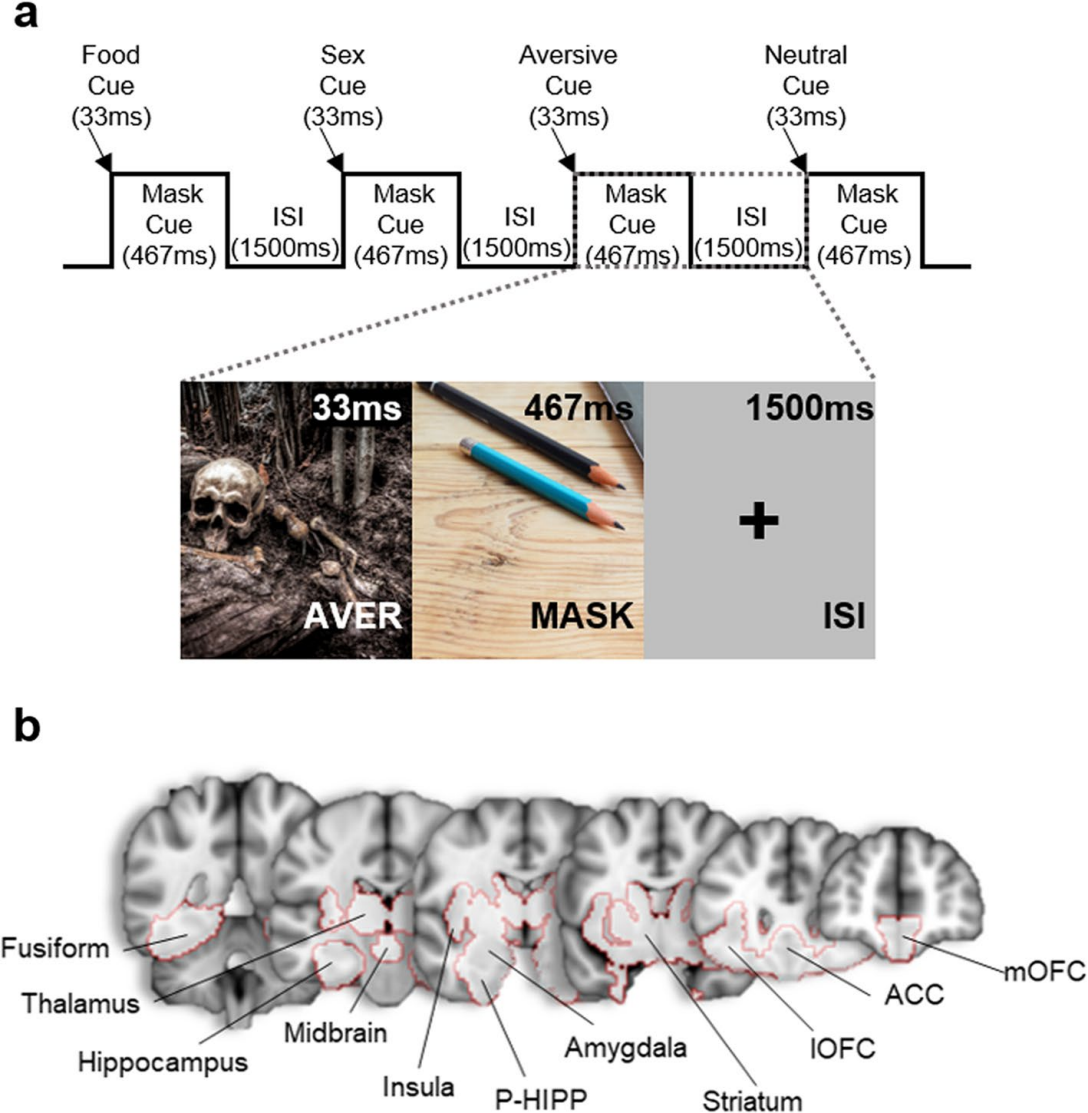
**a** Example images and design of the 33 ms Evocative Cue Task. Number of total trials were: 192 evocative (food, sex, aversive), 192 mask, 48 neutral, and 48 null; duration  of images were: 33 ms evocative; 467 mask; 33 ms neutral; 1500 ms null. **b** Cue-reactive regions: medial (mOFC) and lateral (lOFC) orbitofrontal cortex; anterior cingulate cortex (ACC); striatum; amygdala, parahippocampus (P-HIPP); insula; midbrain; hippocampus; thalamus; and fusiform. Images in (**a**) taken from website, Pexels, and are free-to-use. Image in (**b**) adapted from Regier et al., [43] with permission

**Fig. 2:**
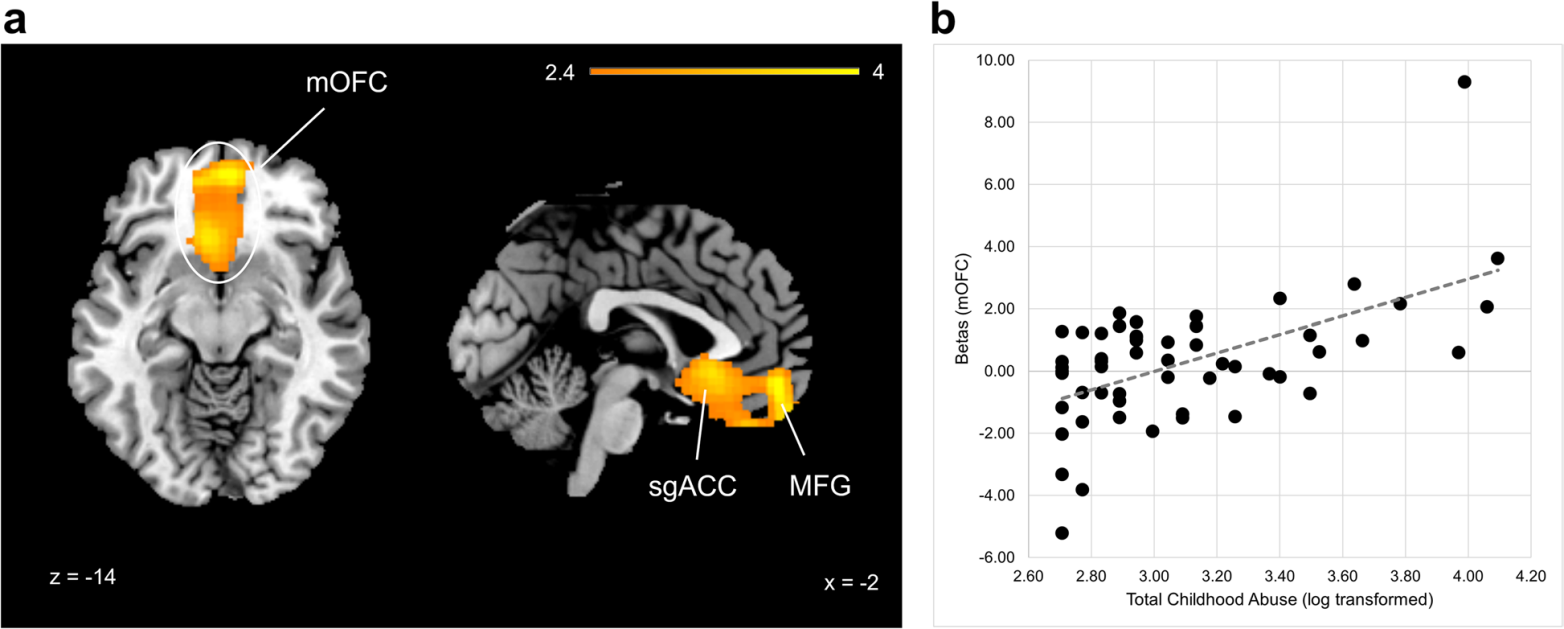
Brain activity in response to 33 ms aversive (vs. neutral) cues, correlated with total childhood abuse scores (TCAs). **A** Brain images showing a significant relationship of brain response to aversive cues with TCAs in the ventral medial orbitofrontal cortex (mOFC), including nodes in the subgenual anterior cingulate cortex (sgACC) and the medial frontal gyrus (MFG). Images shown at 2.4 to 4 for visualization purposes. **B** Scatterplot of the mean beta values extracted from the significant relationship in (**A**) plotted as a function of TCAs (log-transformed due to non-normal distribution). r^2^ value of this relationship was 0.32 (used as the baseline for multiple regression and testing of moderating variables [see Table 3])

Brain data from first-level analyses, both contrast data (e.g., aversive – neutral) and structural data, were examined in association with TCAs and TCNs using SPM. Specifically, individual scans were entered into a one-sample t-test, and then TCAs or TCNs were added as “covariates of interest”, to obtain parameter estimates correlated with increased or decreased brain activity as a function of increased TCAs/TCNs. Due to non-normal distributions, TCAs and TCNs were log-transformed and normalized prior to correlations. Two methods were utilized to reduce false positives and negatives in the results: 1) A “cue-reactive” mask (Regier et al., [43]) limited the number of voxels needed for correction (Fig. 1); 2) Cluster correction was calculated using AFNI’s updated 3dClustSim function [61, 62], utilizing the spatial autocorrelation function (ACF). Voxel-level threshold was set at *p* <  0.005, and cluster-level threshold was set at *p* <  0.005. The cue-reactive mask included a priori anatomical regions of interest (aROIs), previously found to respond to brief, evocative stimuli (Childress et al., 1999, [40,63]; Wetherill et al., 2014 [64]; Regier et al., [42, 43, 55]): amygdala, ventral and dorsal striatum, hippocampus, thalamus, midbrain, ventral medial (mOFC) and lateral orbitofrontal cortex, parahippocampus, occipital cortex, and visual association areas (i.e., fusiform).

For structural data, we analyzed data from the individual a priori regions making up the cue-reactive mask. Data from individual aROIs were extracted and analyzed via basic correlations (Pearson’s) with TCAs and TCNs. For non-normal distributions, data was log-transformed. Results for each set of correlations (e.g., all aROIs regions with TCAs or TCNs) were FDR-corrected (alpha: *p* <  0.05).

**Exploratory**

Individual aROIs that constituted the “cue-reactive” mask (see above) were examined as potential correlations with TCAs/TCNs. To test for confounding factors, a series of partial correlations were conducted with aROI parameter estimates as dependent variables, TCAs/TCNs as the independent variable, and mental health and behavioral variables found to be significant with TCAs and/or TCNs as control variables.

##### Multiple Regression Model

To test for moderating effects of mental health and/or behavioral variables, we fit a multiple regression model with the beta values of relevant brain regions (i.e., areas with significant results from second-level analysis – see above) as our outcome variable. We report adjusted estimates for all parameters from our regression model. The regression model was conducted using MATLAB’s *regress* function, preparing each calculation by creating a matrix that included a row of ones, the outcome variable, the main predictor variable (TCAs/TCNs), and one of the secondary predictor variables (i.e., depression, anxious attachment, sensation seeking, or impulsivity scores). F values were calculated using the following formula:

$$F=\frac{adjR_L^2-{adjR}_S^2/\left({k}_L-{k}_S\right)}{\left(1-{r}_2^2\right)/{df}_L}$$

where df_L_ = n - (p_1_ + k + 1), p_1_ is the *p value* from regression 1, *k* is the number of predictor variables, *L* is the model with more variables, and *S* is the model with fewer variables. r squared values (adjR^2^) were adjusted with the following formula:

$${adjR}^2=1-\frac{\left(n-1\right)}{n-k-1}\ast \left(1-{r}^2\right)$$

If the calculated value of “F” was greater than the critical value obtained from an “F” table with alpha = 0.05, the moderation was considered significant.

## Results

### Sample characteristics

Sixty young adult women participated in the study; the current analyses are based on 54 participants with useable fMRI scans (six were excluded: claustrophobia = 4; motio*n* = 1; heart murmur = 1). The average age of these participants was 20.87 (±2.5) years. The majority of the individuals identified as African American (*n* = 36; 61%), with the remainder identifying as White (*n* = 13, 24%), Hispanic (*n* = 8, 15%), Asian American (*n* = 4, 7%), and American Indian (*n* = 1). In the past 30 days, approximately two-thirds of the participants used alcohol (68%), less than a third used marijuana (32%), and only 5 smoked cigarettes (9%). A history of childhood abuse was self-reported via the CTQ (see methods), with 39% reporting some form of moderate or severe childhood abuse (17% emotional abuse, 24% physical abuse, and 20% sexual abuse) and 19% reporting some form of moderate or severe childhood neglect (15% emotional neglect, 9% physical neglect). Mean (SD) TCAs was 24.1 (11.5) and mean (SD) TCNs was 15.3 (± 6.1).

### Relationships of demographics and health variables with TCA and TCN

Demographic and health variables were evaluated for their relationship with TCAs and TCNs (Table 1). With medium effect sizes, results showed higher TCAs (t(52) = 4.29, *p* <  0.001, CI95: [− 24.0–8.7]) and TCNs (t(52) = 3.59, *p* < 0.001, CI95: [− 11.8–3.3]) in those experiencing physical abuse as an adult. With small effect sizes, results showed higher TCAs in those whose mothers did not finish high school (t(52) = 2.17, *p* = 0.035, CI95 [− 15.6–0.6]) and in those with more food insecurity (t(52) = 2.56, *p* = 0.013, CI95 [− 16.0–1.9]). With small effect sizes, results showed higher TCNs in those with higher BMI (*r* = .33, *p* = 0.022, CI95 [.07 .55]) and history of sexually-transmitted infections (t(52) = 2.17, *p* = 0.035, CI95: [− 6.8–0.3]). Associations of TCAs and TCNs with maladaptive behaviors and mental health symptoms revealed several significant correlations (Table 2). Higher TCAs and TCNs were associated with higher impulsivity scores (TCAs, *r* = .38, *p* = 0.005; TCNs, *r* = .39, *p* = 0.003) and depression symptoms (TCAs, *r* = .56, *p* < 0.001; TCNs, *r* = .31, *p* = 0.021). In contrast, only higher TCAs were associated with higher anxious attachment scores (*r* = .55, p < 0.001).

**Table 1:** Demographic and Health Variables

*n* = 54			Total Abuse Score (TCAs)				Total Neglect Score (TCNs)			
	Mean	SD	Stat*	*P* val**	CI95 (lower)	CI95 (upper)	Stat*	*P* val**	CI95 (lower)	CI95 (upper)
Age	20.87	2.50	*r* = −.17	0.220	−.42	−.10	*r* = −.13	0.360	−.38	.14
Race										
Black	61%		F = 1.65	0.200	–	–	F = .79	0.450	–	–
White	20%									
Asian	6%									
Other***	11%									
BMI (*n* = 47)	25.67	7.64	*r* = .10	0.520	−.17	.36	*r* = .33	**0.022**	.07	.55
Cigarette Use	0.09	0.29	1.05	0.300	−16.4	5.2	0.57	0.572	−7.4	4.1
Alcohol Use	0.69	0.47	0.26	0.793	−5.9	7.7	0.90	0.374	−2.0	5.2
Marijuana Use	0.31	0.47	0.83	0.413	−9.5	4.0	1.19	0.239	−5.7	1.4
Mom finish HS	0.20	0.41	2.16	**0.035**	−15.6	−0.6	1.03	0.308	−6.2	2.0
Food Insecurity	0.25	0.43	2.56	**0.013**	−16.0	− 1.9	0.23	0.820	−3.5	4.4
Forced Sex (adult)	0.19	0.39	1.36	0.181	−13.4	2.6	0.05	0.961	−4.4	4.2
Physical Abuse (adult)	0.15	0.36	4.28	**0.000**	−24.0	−8.7	3.59	**0.001**	−11.8	−3.3
History of STIs	0.41	0.50	0.70	0.490	−8.6	4.2	2.17	**0.035**	−6.8	−0.3
Emotional IPV	0.65	0.48	1.24	0.220	−10.6	2.5	0.93	0.354	−5.1	1.9
Physical IPV	0.35	0.48	1.18	0.244	−10.4	2.7	0.89	0.378	−5.0	1.9
Sexual IPV	0.24	0.43	0.44	0.665	−9.0	5.8	0.58	0.567	−2.8	5.0

*SD* Standard deviation, *CI95* 95% confidence intervals, *P val* p value, *Stat* Statistic, *BMI* Body mass index, *HS* High school, *STIs* Sexually transmitted infections, *IPV* Interpersonal violence

*Stat (statistic value) is “t” value unless otherwise noted

**Table includes unadjusted *p* values with confidence intervals for visualization purposes. FDR-corrected *p* values for TCAs is *p* ≤ 0.013 TCNs ≤0.003 are considered significant

***Other category Includes American Indian, Pacific Islander, and self-identified categories of Hispanic, Taino, Latino, or unspecified

**Table 2:** Behavioral and Mental Health Variables

			Total Abuse Score (TCAs)				Total Neglect Score (TCNs)			
	Mean	SD	R val	CI95 (low)	CI95 (up)	*P* val*	R val	CI95 (low)	CI95 (up)	*P* val*
**Impulsivity**	60.09	11.40	0.38	0.12	0.59	**0.005**	0.39	0.14	0.60	**0.003**
**Anxious Attachment**	7.98	2.69	0.55	0.33	0.71	**< 0.001**	0.28	0.01	0.51	0.042
Sensation Seeking	24.69	5.35	−0.03	−0.30	0.24	0.835	0.07	−0.20	0.33	0.602
**Depression**	17.20	10.94	0.56	0.34	0.72	**< 0.001**	0.31	0.05	0.53	**0.021**

FDR-corrected *p* values for TCAs is *p* ≤ 0.005 TCNs ≤0.021 are considered significant

*SD* Standard deviation, *R val* R value, *CI95* 95% confidence intervals, *P val p* value

*Table includes unadjusted *p* values with confidence intervals for visualization purposes

### Differences in brain response to evocative images as a function of TCA and TCN

#### fMRI: functional

Differences of brain response within the cue-reactive mask (see methods) as a function of childhood maltreatment was assessed via an event-related task featuring presentation of backward-masked 33 msec evocative cues. Young women with higher TCAs had a heightened brain response to aversive (but not romantic or food) minus neutral cues (voxel-level threshold: *p* < 0.005, cluster-level threshold: *p* < 0.005 [k > 761]) in a medial orbitofrontal cortex (mOFC) cluster that included the subgenual anterior cingulate cortex (sgACC) and middle frontal gyrus (MFG) (Fig. 2). There was no significant correlation, after correction, of the brain response to any of the evocative cues with TCNs. For results from correlations of TCAs with all a priori regions composing the “cue-reactive” mask, see Supplementary Fig. 1.

#### fMRI: structural

Though not the primary focus of the study, we analyzed differences in GMV as a function of childhood maltreatment as a replication test of previous studies (see introduction). Results confirmed significant inverse relationships of maltreatment and GMV. Specifically, these unadjusted confidence intervals showed that higher TCAs were significantly associated with lower GMV in several regions, including the amygdala (*r* = −.30 [upper: −.53; lower: = − .034], *p* = 0.034), parahippocampus (*r* = −.38; [lower = −.59; upper = −.12], *p* = 0.006), lateral OFC (*r* = −.36; [lower = −.58; upper = −.09], *p* = 0.01), and mOFC (*r* = −.29; [lower = −.52; upper = −.02], *p* = 0.036). Results from the relationship of *TCNs* with GMV revealed a significant inverse relationship with the thalamus (*r* = −.31; [lower = −.54; upper = −.04], *p* = 0.026). See Supplementary Table 1 for results from all aROIs from the cue-reactive mask.

### Moderating factors of significant relationships found between the functional brain data and TCA/TCN

Follow-up analyses were conducted to assess the effects of mental health and behavioral variables on the relationship between TCAs and the brain response to aversive cues. When controlling for depression symptoms, anxious attachment, and impulsivity, the TCAs/mOFC relationship remained, and in fact it appeared to involve more brain area activity than the TCAs/mOFC relationship alone (Supplementary Fig. 1). The relationship of TCAs and the brain response to aversive cues may even extend beyond the mOFC into other areas, when controlling for impulsivity (e.g., nucleus accumbens [*r* = .34, *p* = 0.014, uncorrected]) (Supplemental Table 2).

Multiple regression was conducted to investigate potential moderating factors of the significant relationship found between the functional brain data and childhood maltreatment. These factors included mental health and behavioral factors found to be significantly associated with higher TCAs (see above and Table 2). Specifically, it was tested whether the significant relationship of a heightened mOFC response to aversive (−neutral) cues with higher TCAs, of which the R^2^ value was 0.32 (Fig. 2), was moderated by depressive symptoms, anxious attachment, and/or impulsivity scores.

Regression results revealed the link between mOFC response to aversive cues and TCAs was significantly moderated by impulsivity (Δr^2^ = .13, F = 13.46, *p* < 0.05) (Table 3), but not depression (Δr^2^ = .01, F = 0.45, NS) or anxious attachment (Δr^2^ = −.01,, NS). Impulsivity’s moderation effect was not simply to strengthen or to reduce the relationship between TCAs and the brain response to aversive cues. Instead, as is often the case for moderator variables, impulsivity scores significantly improved explanation for the *variability* in the relationship of TCAs and the mOFC response to aversive (−neutral) cues. Improvement in the model’s explanatory power is likely due to higher impulsivity scores generally associated with a *reduction* in mOFC response to aversive cues.

**Table 3:** Multiple Regression Results: Moderation effect of mental health and/or behavioral variables on the relationship between mOFC and TCAs

Medial OFC	r Squared	Change in r^2^	F test (*p* value)
Total Abuse (TCAs)	r^2^: .32		
**Impulsivity (BIS)**	**r**^**2**^**: .46 (Adj: .45)**	**.14 (Adj: .13)**	**F Stat = 13.46 (*p*** **< 0.05)**

## Discussion

This study aimed to understand relationships between childhood maltreatment, mental health symptoms and maladaptive behaviors, and brain function. We found that greater severity of childhood abuse (TCAs) but not neglect (TCNs) was associated with a heightened brain response in the mOFC to aversive (but not romantic or food) cues as compared to neutral cues. Higher levels of childhood abuse were associated with elevated scores for several behavioral and mental health factors, including depression, anxious attachment, and impulsivity. Our follow-up analyses found that, among these factors, impulsivity was the only one found to be a significant moderator of the link between a heightened brain response to aversive (−neutral) cues in the mOFC and higher TCAs.

Previous studies have identified an increased response to emotional stimuli associated with maltreatment in the threat detection and response circuit [15], that includes sensory, thalamic, subcortical, and prefrontal cortical regions, including the mOFC. This threat detection network is thought to process both conscious and subconscious stimuli. In this circuit, the mOFC is thought to regulate the response to threat via inhibition of subcortical (e.g., amygdala) activity. Other imaging studies have shown the mOFC is involved with emotional processing [65], and is part of a network, along with subcortical and visual cortical regions, to process and evaluate affective signals [66]. Several studies have highlighted the relationship of maltreatment and increased fMRI response to negative stimuli in subcortical regions [22, 28, 67–69], and previous research has found altered functional connectivity between the mOFC and these regions in those with higher maltreatment [70].

Surprisingly, there were no significant relationships between childhood maltreatment and the brain response to appetitive cues, as had been hypothesized. A previous study from our lab found a positive correlation between prior abuse and subcortical response to rewarding stimuli [42]. One possibility for this outcome is that the rewarding images used in the present study were not salient enough to elicit a differential response. Though we included some romantic images to better match the population (e.g., majority Black), most of the “sexual” images via IAPS are now decades old and predominately for a white audience. Stimuli and population type, and the match between the two, determines to a degree the reactivity to such cues. For example, in our previous study with cocaine patients, we found a robust relationship between prior abuse and heightened mesolimbic activity to drug cues [42]; these cues were specifically designed for this cocaine use disorder population.

In our follow-up analyes, data showed that while higher levels of TCAs and impulsivity were positively correlated (Table 2), higher impulsivity scores seemed to be associated with a reduction in mOFC activity to aversive cues, and when controlling for TCAs, this lower mOFC/higher impulsivity relationship was significant (R = −.44, *p* = 0.007). The positive TCAs/impulsivity relationship, positive TCAs/mOFC relationship, but inverse impulsivity/mOFC relationship may seem conflicting; however, these findings are preceded by previous studies demonstrating similar results. For example, studies have shown that higher impulsivity is associated with decreased reactivity to negative stimuli and situations in several regions, including the OFC [34–37]. These data may partially explain the negative urgency facet of impulsivity [71]. Together, these data suggest two types of responses, depending on the variable: 1) an over-regulation of a subconsciously-processed threat response (i.e., higher mOFC and more TCAs); and 2) an under-regulation to a threat response (i.e., higher mOFC and less impulsivity). More research is needed to understand whether these different responses might be maladaptive or possibly a sign of resilience.

Similar to previous studies [14, 15], the present study showed a reduction of GMV correlated with increased maltreatment; specifically, for higher TCAs, there was a reduction in the OFC, amygdala, and parahippocampus; and for higher TCNs, there was a reduction in the thalamus. In addition, there was a trend of reduced GMV correlated with higher TCAs in the hippocampus (*p* = 0.06), similar to results from several previous studies [22, 25, 26]. Brain structure and function are correlated [72]; thus, structural changes may account for observed functional differences in individuals with childhood maltreatment affects.

Understanding the brain’s responses to evocative stimuli in those who have a history of maltreatment has important implications for future research and practice. Results revealed that emerging adult women with greater prevalence of childhood abuse had heightened mOFC response to “unseen” (33 ms) aversive (−neutral) cues, a relationship that was moderated by higher impulsivity scores. These results can begin to explain behavioral differences of those with maltreatment histories when exposed to triggering or threatening stimuli compared to those who do not have a history of trauma or abuse. These data also highlight the profound biologic impact of trauma, as the aversive cues were not consciously processed by the participants, yet we were still able to see meaningful changes in the brain as a function of maltreatment history. In addition, the discovered moderating role of impulsivity encourages future interventions to support emotion regulation and distress management in this population, as previous literature has found an association of negative emotional states along with emotional dysregulation with impulsivity [73–75]. Based on the literature and our own correlations between maltreatment and behavioral/mental health variables, we have interpreted the heightened activation of the mOFC as a ‘vulnerability’ signature. However, prospective designs can be used to confirm whether this brain response indeed indicates vulnerability (to development of diagnosable clinical disorders of anxiety, etc.), or whether it might be a marker of ‘resilience’, indicating the brain’s reflexive but effective coping with the aversive stimuli (even outside awareness). In addition, future research could examine this population’s ability to explicitly regulate their subjective and brain response to aversive cues, to better understand how these brain responses are linked to behavioral choices after exposure to threatening stimuli.

### Limitations

While this study had clear results, caution should be used when translating these findings outside the study population, which consisted of emerging adult women (ages 18–24). In addition, we did not have data on trauma characteristics outside of maltreatment type and were unable to measure the impact of the trauma on the participant’s daily life, potentially missing additional moderating variables that may have contributed to the participants’ brain responses. Though efforts were made to ensure accurate reporting (e.g., computerized task, with assurances that answers were confidential), behavioral and trauma measures were self-reported. Some data has suggested that self-report and objective measures differ (e.g., self-reported impulsivity vs behavioral impulsivity [e.g., [74]). Finally, the GMV data was only able to utilize 51 out of 54 subjects due to data loss. Despite these limitations, this study was the first of its kind to describe the relationships between childhood maltreatment, mental health symptoms and maladaptive behaviors, and brain structure/function in adult women. Results of this study underscore the impact of trauma and violence in emerging adult women at the level of the brain and can offer a foundation for future interventions to mitigate distress in this population.

## Conclusion

Emerging adult women with higher incidences of childhood emotional, physical, and sexual abuse had more mental health symptoms and maladaptive behaviors. Higher levels of childhood abuse were also associated with elevated mOFC activity to 33 ms aversive cues, backward-masked, and thus presented outside conscious awareness. The follow-up analyses found that impulsivity had a complex moderating effect on the relationship between childhood abuse and the brain response to 33 ms aversive cues, suggesting conflicting signals of under- and over-regulation of negative stimuli. These data may help guide the treatment of those with childhood abuse, targeting impulse control and emotional regulation with behavioral and/or biological supports.

## Supplementary Information

**Additional file 1: Supplementary Table 1.** Grey matter volume (GMV) associated with childhood maltreatment. **Supplementary Table 2.** Relationship of TCAs with mOFC response, controlling for mental health and behavioral variables. **Supplemental Fig. 1.** Overlays of brain response to aversive (−neutral) cues while either controlling or not controlling for impulsivity. Yellow overlay shows the brain response correlated with TCAs, while controlling for impulsivity. Red overlay represents overlap of brain response to aversive (−neutral) cues while either not controlling for impulsivity. Evidence suggests that when controlling for impulsivity, brain activity extends beyond those regions (e.g., to ventral striatum) that are associated with TCAs alone, while also providing better coverage (i.e., fills in the “gaps” in mOFC).

## Data Availability

All data and materials used in the current study are available upon request.

## References

[1] Butchart A, World Health Organization, International Society for the Prevention of Child Abuse and Neglect (2006). Preventing child maltreatment: a guide to taking action and generating evidence.

[2] Stoltenborgh M, Bakermans-Kranenburg MJ, Alink LRA, van IJzendoorn MH (2015). The Prevalence of Child Maltreatment across the Globe: Review of a Series of Meta-Analyses. Child Abuse Rev.

[3] Scher CD, Forde DR, McQuaid JR, Stein MB (2004). Prevalence and demographic correlates of childhood maltreatment in an adult community sample. Child Abuse Negl.

[4] Finkelhor D, Turner HA, Shattuck A, Hamby SL (2013). Violence, crime, and abuse exposure in a National Sample of children and youth: an update. JAMA Pediatr.

[5] Rodgers CS, Lang AJ, Laffaye C, Satz LE, Dresselhaus TR, Stein MB (2004). The impact of individual forms of childhood maltreatment on health behavior. Child Abuse Negl.

[6] Corso PS, Edwards VJ, Fang X, Mercy JA (2008). Health-related quality of life among adults who experienced maltreatment during childhood. Am J Public Health.

[7] Gilbert R, Widom CS, Browne K, Fergusson D, Webb E, Janson S (2009). Burden and consequences of child maltreatment in high-income countries. Lancet Lond Engl.

[8] Green JG, McLaughlin KA, Berglund PA, Gruber MJ, Sampson NA, Zaslavsky AM (2010). Childhood adversities and adult psychopathology in the National Comorbidity Survey Replication (NCS-R) I: associations with first onset of DSM-IV disorders. Arch Gen Psychiatry.

[9] Scott KM, McLaughlin KA, Smith DAR, Ellis PM (2012). Childhood maltreatment and DSM-IV adult mental disorders: comparison of prospective and retrospective findings. Br J Psychiatry.

[10] Liu RT (2019). Childhood maltreatment and impulsivity: Meta-analysis and recommendations for future study. J Abnorm Child Psychol.

[11] Baer JC, Martinez CD (2006). Child maltreatment and insecure attachment: a meta-analysis. J Reprod Infant Psychol.

[12] Muller RT, Sicoli LA, Lemieux KE (2000). Relationship between attachment style and posttraumatic stress symptomatology among adults who report the experience of childhood abuse. J Trauma Stress.

[13] Shonkoff JP, Garner AS, Siegel BS, Dobbins MI, Earls MF, The Committee on Psychosocial Aspects of Child and Family Health C on EC (2012). The lifelong effects of early childhood adversity and toxic stress. Pediatrics..

[14] Hart H, Rubia K (2012). Neuroimaging of child abuse: a critical review. Front Hum Neurosci.

[15] Teicher MH, Samson JA, Anderson CM, Ohashi K (2016). The effects of childhood maltreatment on brain structure, function and connectivity. Nat Rev Neurosci.

[16] Putnam FW (2006). The impact of trauma on child development. Juv Fam Court J.

[17] Badr HE, Naser J, Al-Zaabi A, Al-Saeedi A, Al-Munefi K, Al-Houli S (2018). Childhood maltreatment: A predictor of mental health problems among adolescents and young adults. Child Abuse Negl.

[18] Edwards VJ, Holden GW, Felitti VJ, Anda RF (2003). Relationship between multiple forms of childhood maltreatment and adult mental health in community respondents: results from the adverse childhood experiences study. Am J Psychiatry.

[19] Taber-Thomas B, Pérez-Edgar K. Emerging adulthood brain development. The Oxford handbook of emerging adulthood. 2015. https://www.oxfordhandbooks.com/view/10.1093/oxfordhb/9780199795574.001.0001/oxfordhb-9780199795574-e-15. Accessed 22 Mar 2021.

[20] Bremner JD, Vythilingam M, Vermetten E, Southwick SM, McGlashan T, Nazeer A (2003). MRI and PET study of deficits in hippocampal structure and function in women with childhood sexual abuse and posttraumatic stress disorder. Am J Psychiatry.

[21] Carrion VG, Garrett A, Menon V, Weems CF, Reiss AL (2008). Posttraumatic stress symptoms and brain function during a response-inhibition task: an fMRI study in youth. Depress Anxiety.

[22] Dannlowski U, Stuhrmann A, Beutelmann V, Zwanzger P, Lenzen T, Grotegerd D (2012). Limbic scars: long-term consequences of childhood maltreatment revealed by functional and structural magnetic resonance imaging. Biol Psychiatry.

[23] De Brito SA, Essi V, Sebastian CL, Kelly PA, Andrea M, Helen M (2012). Reduced orbitofrontal and temporal grey matter in a community sample of maltreated children. J Child Psychol Psychiatry.

[24] Ahmed F, Spottiswoode BS, Carey PD, Stein DJ, Seedat S (2012). Relationship between Neurocognition and regional brain volumes in traumatized adolescents with and without posttraumatic stress disorder. Neuropsychobiology..

[25] Dillon DG, Holmes AJ, Birk JL, Brooks N, Lyons-Ruth K, Pizzagalli DA (2009). Childhood adversity is associated with left basal ganglia dysfunction during reward anticipation in adulthood. Biol Psychiatry.

[26] Bremner JD, Narayan M, Staib LH, Southwick SM, McGlashan T, Charney DS (1999). Neural correlates of memories of childhood sexual abuse in women with and without posttraumatic stress disorder. Am J Psychiatry.

[27] Maheu FS, Dozier M, Guyer AE, Mandell D, Peloso E, Poeth K (2010). A preliminary study of medial temporal lobe function in youths with a history of caregiver deprivation and emotional neglect. Cogn Affect Behav Neurosci.

[28] Dannlowski U, Kugel H, Huber F, Stuhrmann A, Redlich R, Grotegerd D (2013). Childhood maltreatment is associated with an automatic negative emotion processing bias in the amygdala. Hum Brain Mapp.

[29] Goff B, Gee DG, Telzer EH, Humphreys KL, Gabard-Durnam L, Flannery J (2013). Reduced nucleus Accumbens reactivity and adolescent depression following early-life stress. Neuroscience..

[30] Boecker R, Holz NE, Buchmann AF, Blomeyer D, Plichta MM, Wolf I (2014). Impact of early life adversity on reward processing in young adults: EEG-fMRI results from a prospective study over 25 years. PLoS One.

[31] Hanson JL, Hariri AR, Williamson DE (2015). Blunted ventral striatum development in adolescence reflects emotional neglect and predicts depressive symptoms. Biol Psychiatry.

[32] Russo SJ, Nestler EJ (2013). The brain reward circuitry in mood disorders. Nat Rev Neurosci.

[33] Pizzagalli DA (2014). Depression, stress, and Anhedonia: toward a synthesis and integrated model. Annu Rev Clin Psychol.

[34] Brown MRG, Benoit JRA, Juhás M, Lebel RM, MacKay M, Dametto E (2015). Neural correlates of high-risk behavior tendencies and impulsivity in an emotional go/NoGo fMRI task. Front Syst Neurosci.

[35] Coccaro EF, McCloskey MS, Fitzgerald DA, Phan KL (2007). Amygdala and orbitofrontal reactivity to social threat in individuals with impulsive aggression. Biol Psychiatry.

[36] Plichta MM, Scheres A (2014). Ventral–striatal responsiveness during reward anticipation in ADHD and its relation to trait impulsivity in the healthy population: A meta-analytic review of the fMRI literature. Neurosci Biobehav Rev.

[37] Lemiere J, Danckaerts M, Van Hecke W, Mehta MA, Peeters R, Sunaert S (2012). Brain activation to cues predicting inescapable delay in adolescent attention deficit/hyperactivity disorder: an fMRI pilot study. Brain Res.

[38] Corral-Frías NS, Nikolova YS, Michalski LJ, Baranger D, a. A, Hariri AR, Bogdan R. (2015). Stress-related anhedonia is associated with ventral striatum reactivity to reward and transdiagnostic psychiatric symptomatology. Psychol Med.

[39] Bernstein DP, Fink L, Handelsman L, Foote J, Lovejoy M, Wenzel K (1994). Initial reliability and validity of a new retrospective measure of child abuse and neglect. Am J Psychiatry.

[40] Childress AR, Ehrman RN, Wang Z, Li Y, Sciortino N, Hakun J (2008). Prelude to passion: limbic activation by “unseen” drug and sexual cues. PLoS One.

[41] Morris JS, Ohman A, Dolan RJ (1998). Conscious and unconscious emotional learning in the human amygdala. Nature..

[42] Regier PS, Monge ZA, Franklin TR, Wetherill RR, Teitelman A, Jagannathan K, et al. Emotional, physical and sexual abuse are associated with a heightened limbic response to cocaine cues. Addict Biol. 2016. 10.1111/adb.12445. 10.1111/adb.12445 PMC5767126 27654662

[43] Regier PS, Teitelman AM, Jagannathan K, Monge ZA, McCondochie C, Elkind J (2020). Women at greater sexual risk for STIs/HIV have a lower mesolimbic and affective Bias response to sexual stimuli. Front. Behav Neurosci.

[44] Morrison-Beedy D, Carey MP, Tu X (2006). Accuracy of audio computer-assisted self-interviewing (ACASI) and self-administered questionnaires for the assessment of sexual behavior. AIDS Behav.

[45] Bernstein DP, Stein JA, Newcomb MD, Walker E, Pogge D, Ahluvalia T (2003). Development and validation of a brief screening version of the childhood trauma questionnaire. Child Abuse Negl.

[46] Edmiston EE, Wang F, Mazure CM, Guiney J, Sinha R, Mayes LC (2011). Corticostriatal-limbic gray matter morphology in adolescents with self-reported exposure to childhood maltreatment. Arch Pediatr Adolesc Med.

[47] Frodl T, Reinhold E, Koutsouleris N, Reiser M, Meisenzahl EM (2010). Interaction of childhood stress with hippocampus and prefrontal cortex volume reduction in major depression. J Psychiatr Res.

[48] Teicher MH, Dumont NL, Ito Y, Vaituzis C, Giedd JN, Andersen SL (2004). Childhood neglect is associated with reduced corpus callosum area. Biol Psychiatry.

[49] Radloff LS (1977). The CES-D scale: A self-report depression scale for research in the general population. Appl Psychol Meas.

[50] Patton JH, Stanford MS, Barratt ES (1995). Factor structure of the Barratt impulsiveness scale. J Clin Psychol.

[51] Collins NL, Read SJ (1990). Adult attachment, working models, and relationship quality in dating couples. J Pers Soc Psychol.

[52] Kershaw TS, Milan S, Westdahl C, Lewis J, Rising SS, Fletcher R (2007). Avoidance, anxiety, and sex: the influence of romantic attachment on HIV-risk among pregnant women. AIDS Behav.

[53] Hoyle RH, Stephenson MT, Palmgreen P, Lorch EP, Donohew RL (2002). Reliability and validity of a brief measure of sensation seeking. Personal Individ Differ.

[54] Zuckerman M, Neeb M (1979). Sensation seeking and psychopathology. Psychiatry Res.

[55] Regier PS, Jagannathan K, Franklin TR, Wetherill RR, Langleben DD, Gawyrsiak M (2021). Sustained brain response to repeated drug cues is associated with poor drug-use outcomes. Addict Biol.

[56] Lang PJ, Bradley MM, Cuthbert BN. International affective picture system (IAPS): Instruction manual and affective ratings. The center for research in psychophysiology, University of Florida. 1999.

[57] Benjamini Y, Hochberg Y (1995). Controlling the false discovery rate: A practical and powerful approach to multiple testing. J R Stat Soc Ser B Methodol.

[58] Groppe D (2020). fdr_bh (). MATLAB central file exchange.

[59] MATLAB. Version 9.6.0.1135713 (R2019a). Natick: The MathWorks Inc.; 2019.

[60] Franklin TR, Acton PD, Maldjian JA, Gray JD, Croft JR, Dackis CA (2002). Decreased gray matter concentration in the insular, orbitofrontal, cingulate, and temporal cortices of cocaine patients. Biol Psychiatry.

[61] Cox RW, Chen G, Glen DR, Reynolds RC, Taylor PA (2017). FMRI clustering in AFNI: false-positive rates Redux. Brain Connect.

[62] Cox RW, Chen G, Glen DR, Reynolds RC, Taylor PA (2017). fMRI clustering and false-positive rates. Proc Natl Acad Sci U S A.

[63] Childress AR, Mozley PD, McElgin W, Fitzgerald J, Reivich M, O’Brien CP. Limbic activation during cue-induced cocaine craving. Am J Psychiatry. 1999;156:11–8. 10.1176/ajp.156.1.11 PMC2820826 9892292

[64] Wetherill RR, Childress AR, Jagannathan K, Bender J, Young KA, Suh JJ, et al. Neural responses to subliminally presented cannabis and other emotionally evocative cues in cannabis-dependent individuals. Psychopharmacology (Berl). 2014;231:1397–407. 10.1007/s00213-013-3342-z PMC6218642 24186078

[65] Sabatinelli D, Fortune EE, Li Q, Siddiqui A, Krafft C, Oliver WT (2011). Emotional perception: Meta-analyses of face and natural scene processing. NeuroImage..

[66] Murray EA (2007). The amygdala, reward and emotion. Trends Cogn Sci.

[67] Grant MM, Cannistraci C, Hollon SD, Gore J, Shelton R (2011). Childhood trauma history differentiates amygdala response to sad faces within MDD. J Psychiatr Res.

[68] McCrory EJ, De Brito SA, Sebastian CL, Mechelli A, Bird G, Kelly PA (2011). Heightened neural reactivity to threat in child victims of family violence. Curr Biol CB.

[69] van Harmelen A-L, van Tol M-J, Demenescu LR, van der Wee NJA, Veltman DJ, Aleman A (2013). Enhanced amygdala reactivity to emotional faces in adults reporting childhood emotional maltreatment. Soc Cogn Affect Neurosci.

[70] Herringa RJ, Birn RM, Ruttle PL, Burghy CA, Stodola DE, Davidson RJ (2013). Childhood maltreatment is associated with altered fear circuitry and increased internalizing symptoms by late adolescence. Proc Natl Acad Sci.

[71] Wardell JD, Strang NM, Hendershot CS (2016). Negative urgency mediates the relationship between childhood maltreatment and problems with alcohol and cannabis in late adolescence. Addict Behav.

[72] Teicher MH, Samson JA (2013). Childhood maltreatment and psychopathology: A case for Ecophenotypic variants as clinically and Neurobiologically distinct subtypes. Am J Psychiatry.

[73] Schreiber LRN, Grant JE, Odlaug BL (2012). Emotion regulation and impulsivity in young adults. J Psychiatr Res.

[74] Jakubczyk A, Trucco EM, Kopera M, Kobyliński P, Suszek H, Fudalej S (2018). The association between impulsivity, emotion regulation, and symptoms of alcohol use disorder. J Subst Abus Treat.

[75] Malesza M (2019). Stress and delay discounting: the mediating role of difficulties in emotion regulation. Personal Individ Differ.

