# Supplementary material for: In young women, a link between childhood abuse and subliminal processing of aversive cues is moderated by impulsivity
Source: BMC Psychiatry. 2022 Mar 2;22:159. doi: 10.1186/s12888-022-03770-0 (PMC8889687; doi:10.1186/s12888-022-03770-0)
Supplement: Supplementary file 1 — Additional file 1: Supplementary Table 1. Grey matter volume (GMV) associated with childhood maltreatment. Supplementary Table 2. Relationship of TCAs with mOFC response, controlling for mental health and behavioral variables. Supplemental Fig. 1. Overlays of brain response to aversive (−neutral) cues while either controlling or not controlling for impulsivity. Yellow overlay shows the brain response correlated with TCAs, while controlling for impulsivity. Red overlay represents overlap of brain response to aversive (−neutral) cues while either not controlling for impulsivity. Evidence suggests that when controlling for impulsivity, brain activity extends beyond those regions (e.g., to ventral striatum) that are associated with TCAs alone, while also providing better coverage (i.e., fills in the “gaps” in mOFC). [file 12888_2022_3770_MOESM1_ESM.docx]

Supplementary Table 1. Grey matter volume (GMV) associated with childhood maltreatment.

|  | Total Childhood Abuse (TCAs) | | | |  | Total Childhood Neglect (TCNs) | | | |
| --- | --- | --- | --- | --- | --- | --- | --- | --- | --- |
| Region | R value | lower | upper | p value | Region | R value | lower | upper | p value |
| **Amygdala (right)** | **-0.30** | **-0.53** | **-0.03** | **0.034** | Amygdala (right) | -0.12 | -0.38 | 0.16 | 0.386 |
| **OFC (lateral)** | **-0.36** | **-0.58** | **-0.09** | **0.010** | OFC (lateral) | -0.08 | -0.35 | 0.20 | 0.587 |
| **P-HIPP (right)** | **-0.38** | **-0.59** | **-0.12** | **0.006** | P-HIPP (right) | -0.17 | -0.43 | 0.11 | 0.221 |
| **OFC (caudal-medial)** | **-0.29** | **-0.52** | **-0.02** | **0.036** | OFC (caudal-medial) | -0.03 | -0.30 | 0.25 | 0.808 |
| Thalamus | -0.18 | -0.43 | 0.10 | 0.208 | **Thalamus** | **-0.31** | **-0.54** | **-0.04** | **0.026** |
| *Amygdala*  *(left)* | *-0.27* | *-0.51* | *0.01* | *0.059* | Amygdala (left) | -0.11 | -0.37 | 0.17 | 0.437 |
| *Hippocamp. (right)* | *-0.26* | *-0.50* | *0.02* | *0.065* | Hipp (right) | -0.16 | -0.42 | 0.12 | 0.262 |
| *P-HIPP (left)* | *-0.27* | *-0.51* | *0.01* | *0.055* | P-HIPP (left) | -0.14 | -0.40 | 0.14 | 0.317 |
| ACC | -0.14 | -0.40 | 0.14 | 0.326 | ACC | -0.01 | -0.28 | 0.27 | 0.958 |
| Caudate | -0.11 | -0.37 | 0.17 | 0.441 | Caudate | -0.21 | -0.46 | 0.07 | 0.144 |
| Hippocamp. (left) | -0.19 | -0.44 | 0.09 | 0.175 | Hipp (left) | -0.14 | -0.40 | 0.14 | 0.331 |
| Insula | -0.22 | -0.47 | 0.06 | 0.115 | Insula | -0.12 | -0.38 | 0.16 | 0.414 |
| Fusiform | -0.05 | -0.32 | 0.23 | 0.726 | OCC/Fusi | -0.08 | -0.35 | 0.20 | 0.594 |
| PCC | -0.15 | -0.41 | 0.13 | 0.279 | PCC | -0.08 | -0.35 | 0.20 | 0.555 |
| Putamen | -0.04 | -0.31 | 0.24 | 0.761 | Putamen | -0.10 | -0.37 | 0.18 | 0.469 |

Uncorrected results for a priori regions composing the cue-reactive mask. Bolded emphasis indicates a significant relationship (p < 0.05). Italics indicate trends (p < 0.1). OFC: orbitofrontal cortex, P-HIPP: parahippocampus, ACC: anterior cingulate cortex, PCC: posterior cingulate cortex

Supplementary Table 2. Relationship of TCAs with mOFC response, controlling for mental health and behavioral variables

|  | Control Variables for the Relationship between TCAs and mOFC Response to Aversive Cues | | | | | | | | | |
| --- | --- | --- | --- | --- | --- | --- | --- | --- | --- | --- |
| Mask Regions | None | Anxious Att. | Depr. | Impuls. | All | None | Anxious Att. | Depr. | Impuls. | All |
|  | R values (Pearson’s Correlation) | | | | | P values | | | | |
| **NAcc** | **0.28** | **0.27** | **0.29** | **0.34** | **0.31** | **0.040** | **0.050** | **0.033** | **0.014** | **0.027** |
| **Caudal OFC** | 0.15 | *0.23* | *0.26* | 0.19 | **0.29** | 0.269 | *0.097* | *0.059* | 0.164 | **0.039** |
| **mOFC** | **0.46** | **0.44** | **0.53** | **0.58** | **0.56** | **0.000** | **0.001** | **0.000** | **0.000** | **0.000** |
| *Fusiform* | -0.17 | -0.17 | *-0.25* | -0.19 | -0.23 | 0.226 | 0.216 | *0.067* | 0.179 | *0.097* |
| ACC | 0.02 | 0.12 | 0.06 | 0.01 | 0.11 | 0.889 | 0.392 | 0.667 | 0.969 | 0.452 |
| Amygdala | 0.00 | 0.05 | 0.03 | 0.02 | 0.06 | 0.983 | 0.733 | 0.839 | 0.875 | 0.695 |
| Caudate | -0.01 | -0.01 | -0.01 | 0.00 | 0.00 | 0.929 | 0.969 | 0.915 | 0.982 | 0.981 |
| Hippocamp. | -0.10 | -0.12 | -0.13 | -0.10 | -0.13 | 0.452 | 0.405 | 0.351 | 0.465 | 0.366 |
| Insula | -0.12 | 0.01 | -0.06 | -0.15 | -0.01 | 0.371 | 0.962 | 0.665 | 0.271 | 0.941 |
| lateral OFC | -0.04 | 0.07 | 0.06 | -0.03 | 0.10 | 0.755 | 0.602 | 0.669 | 0.835 | 0.467 |
| PCC | 0.08 | 0.06 | 0.04 | 0.13 | 0.06 | 0.545 | 0.665 | 0.788 | 0.354 | 0.679 |
| Parahipp. | -0.08 | -0.04 | -0.11 | -0.06 | -0.07 | 0.558 | 0.775 | 0.416 | 0.688 | 0.646 |
| Putamen | -0.07 | 0.00 | -0.02 | -0.09 | 0.00 | 0.621 | 0.977 | 0.886 | 0.529 | 0.992 |
| STG | -0.08 | -0.02 | -0.08 | -0.10 | -0.04 | 0.544 | 0.898 | 0.588 | 0.469 | 0.766 |
| Thalamus | -0.01 | -0.05 | -0.12 | -0.01 | -0.11 | 0.936 | 0.706 | 0.376 | 0.951 | 0.448 |

Uncorrected results for a priori regions composing the cue-reactive mask. Bolded numbers indicate significant relationship(s). Italics indicate trends. Anxious Att: anxious attachment; Depr: CES-D scores; Impuls: BIS scores


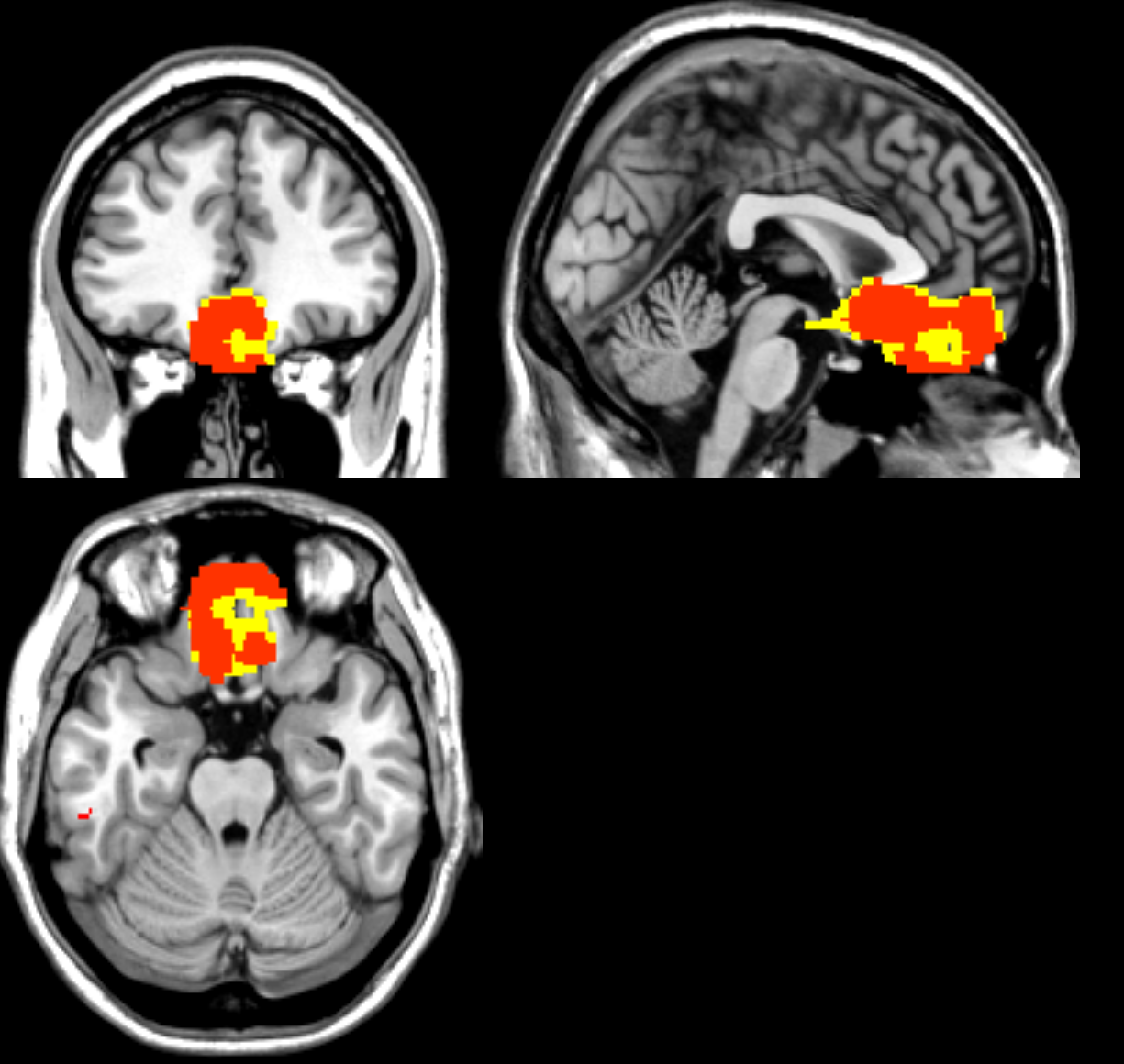


Supplemental Figure 1. Overlays of brain response to aversive (-neutral) cues while either controlling or not controlling for impulsivity. Yellow overlay shows the brain response correlated with TCAs, while controlling for impulsivity. Red overlay represents overlap of brain response to aversive (-neutral) cues while either not controlling for impulsivity. Evidence suggests that when controlling for impulsivity, brain activity extends beyond those regions (e.g., to ventral striatum) that are associated with TCAs alone, while also providing better coverage (i.e., fills in the “gaps” in mOFC).
